# Prediction of risk for early or very early preterm births using high-resolution urinary metabolomic profiling

**DOI:** 10.1186/s12884-024-06974-2

**Published:** 2024-11-25

**Authors:** Yaqi Zhang, Karl G. Sylvester, Ronald J. Wong, Yair J. Blumenfeld, Kuo Yuan Hwa, C. James Chou, Sheeno Thyparambil, Weili Liao, Zhi Han, James Schilling, Bo Jin, Ivana Marić, Nima Aghaeepour, Martin S. Angst, Brice Gaudilliere, Virginia D. Winn, Gary M. Shaw, Lu Tian, Ruben Y. Luo, Gary L. Darmstadt, Harvey J. Cohen, David K. Stevenson, Doff B. McElhinney, Xuefeng B. Ling

**Affiliations:** 1 College of Automation, Guangdong Polytechnic Normal University, Guangzhou, 510665 China; 2 Department of Surgery, Stanford University School of Medicine, Stanford, CA 94305 USA; 3 Department of Pediatrics, Stanford University School of Medicine, Stanford, CA 94305 USA; 4 Department of Obstetrics and Gynecology, Stanford University School of Medicine, Stanford, CA 94305 USA; 5 Center for Biomedical Industry, National Taipei University of Technology, Taipei, 10608 Taiwan; 6 mProbe Inc., Palo Alto, CA 94303 USA; 7 Department of Anesthesiology, Perioperative and Pain Medicine, Stanford University School of Medicine, Stanford, CA 94303 USA; 8 Departments of Cardiothoracic Surgery, Stanford University School of Medicine, Stanford, CA 94305 USA

**Keywords:** Early pregnancy, Preterm risk prediction, Spontaneous preterm birth, Biomarker, Urinary metabolite, LC-MS/MS

## Abstract

**Background:**

Preterm birth (PTB) is a serious health problem. PTB complications is the main cause of death in infants under five years of age worldwide. The ability to accurately predict risk for PTB during early pregnancy would allow early monitoring and interventions to provide personalized care, and hence improve outcomes for the mother and infant.

**Objective:**

This study aims to predict the risks of early preterm (< 35 weeks of gestation) or very early preterm (≤ 26 weeks of gestation) deliveries by using high-resolution maternal urinary metabolomic profiling in early pregnancy.

**Design:**

A retrospective cohort study was conducted by two independent preterm and term cohorts using high-density weekly urine sampling. Maternal urine was collected serially at gestational weeks 8 to 24. Global metabolomics approaches were used to profile urine samples with high-resolution mass spectrometry. The significant features associated with preterm outcomes were selected by Gini Importance. Metabolite biomarker identification was performed by liquid chromatography tandem mass spectrometry (LCMS-MS). XGBoost models were developed to predict early or very early preterm delivery risk.

**Setting and participants:**

The urine samples included 329 samples from 30 subjects at Stanford University, CA for model development, and 156 samples from 24 subjects at the University of Alabama, Birmingham, AL for validation.

**Results:**

12 metabolites associated with PTB were selected and identified for modelling among 7,913 metabolic features in serial-collected urine samples of pregnant women. The model to predict early PTB was developed using a set of 12 metabolites that resulted in the area under the receiver operating characteristic (AUROCs) of 0.995 (95% CI: [0.992, 0.995]) and 0.964 (95% CI: [0.937, 0.964]), and sensitivities of 100% and 97.4% during development and validation testing, respectively. Using the same metabolites, the very early PTB prediction model achieved AUROCs of 0.950 (95% CI: [0.878, 0.950]) and 0.830 (95% CI: [0.687, 0.826]), and sensitivities of 95.0% and 60.0% during development and validation, respectively.

**Conclusion:**

Models for predicting risk of early or very early preterm deliveries were developed and tested using metabolic profiling during the 1st and 2nd trimesters of pregnancy. With patient validation studies, risk prediction models may be used to identify at-risk pregnancies prompting alterations in clinical care, and to gain biological insights of preterm birth.

**Supplementary Information:**

The online version contains supplementary material available at 10.1186/s12884-024-06974-2.

## Introduction

Preterm birth (PTB: delivery before 37 weeks’ gestation) is a serious pregnancy health problem [1, 2]. PTB complications are the main cause of death in infants [3–5]. The risk of PTB is mainly based on clinical criteria, physical examination, measurement of cervical length (CL) with transvaginal ultrasound (TVUS) during the 2nd or 3rd trimester [6, 7]. However, risk stratification at a later stage in pregnancy is not instrumental to guide early intervention. To improve prenatal care and optimize the clinical benefits of prenatal screening tests, early identification of pregnant women at risk for PTB is warranted.

Recent machine-learning techniques have attempted to construct models to predict during pregnancy the risk of having PTB [8, 9]. These prediction models were developed using pre-defined clinical risk factors (psychosocial, sociodemographic, and medical) [10–12] or variables from electronic health records (EHRs) [13]. Molecular biomarkers, including maternal circulating transcripts, proteins, and metabolites, have been identified, which are associated with fetal growth, gestational age (GA) dating, and PTB outcome [14–24]. We have performed maternal serological metabolomic profiling [21] to develop metabolic-pathway-based models to assess fetal GA and predict risk of PTB, which outperformed a commercialized predictor (IBP4/SHBG ratio [25]). Such findings have revealed the potential of using low-cost and rapid serological tests as methods to predict risk of PTB, especially in low-resource areas where ultrasound measurements are unreliable and cost-prohibitive.

Because 70% of urinary molecular content originates from the kidneys and urinary tracts with the remaining 30% from the circulation in healthy individuals, molecular profiling of the urinary content [26, 27] can provide insights into both renal and systemic dysfunctions. Our recent study characterized the weekly baseline profile of the human urinary metabolome during pregnancy, which provides a high-resolution molecular reference [22] for future studies of adverse pregnancy outcomes. In this study, we hypothesized that aberrations from the normal urinary metabolomic profile may identify those pregnancies at risk for PTB. Our findings suggest that measurements of urinary metabolomic biomarkers may serve as a noninvasive, cost-effective, and robust approach for PTB prediction during the 1st or 2nd trimester.

## Methods

### PTB definition

In this study, a healthy full-term pregnancy was defined as a pregnancy ending with a delivery at ≥ 37 weeks’ GA without known complications. PTB was subcategorized into two types: early preterm (< 35 weeks’ GA) and very early preterm (≤ 26 weeks’ GA) spontaneous (i.e., not medically induced) deliveries.

### Cohort construction

30 (19 full-term, 11 preterm) mothers who delivered at Stanford University (SU, Stanford, CA) were recruited to develop the PTB model, and 24 (12 full-term, 12 preterm) mothers who delivered at the University of Alabama, Birmingham (UAB) were recruited and used to validate the model. All enrolled patients had singleton pregnancies. Pregnant women diagnosed with preeclampsia were excluded. This research was done without patient involvement. Patients were not invited to comment on the study design and were not consulted to develop patient relevant outcomes or interpret the results. Patients were not invited to contribute to the writing or editing of this document for readability or accuracy.

### Samples

**Fig. 1:**
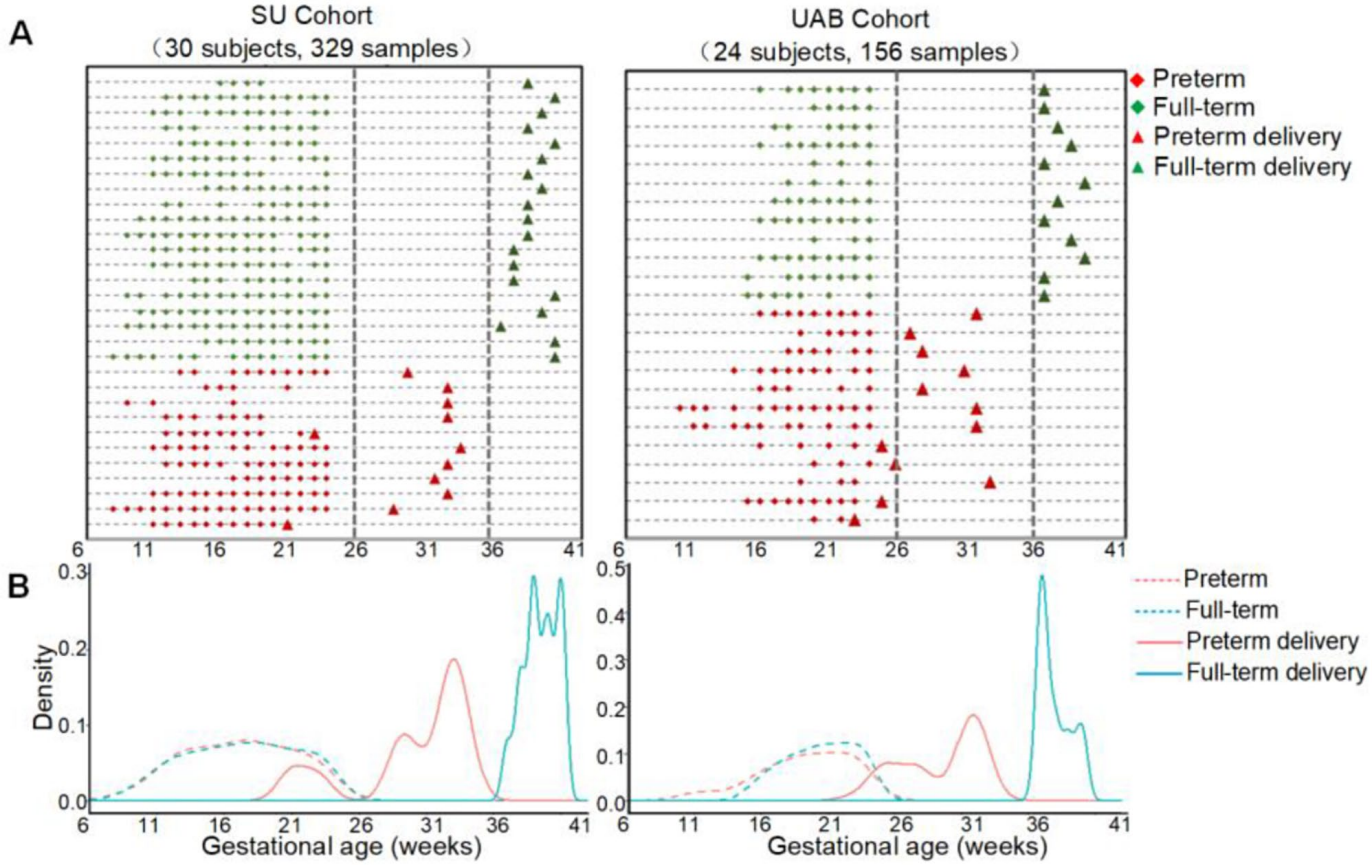
The sample distributions in the two cohorts. (**A**) Charts of urine collection timepoints for Stanford University (SU) and University of Alabama at Birmingham (UAB) cohorts. Each line represents an individual patient. Diamonds and triangles indicate sample collection and delivery dates, respectively. (**B**) Distributions of sample collection (dashed lines) and delivery (solid lines) times for full-term (blue) and very and late preterm (red) pregnancies in the SU and UAB cohorts

Using a previously described urine collection protocol [27], urine samples (*n* = 485) were collected longitudinally from 54 pregnant women. All these samples were gathered from the midstream of fasting urine in the morning. These pregnant women represented a diverse range of races, ethnicities, geographic locations, and socioeconomic backgrounds. The distributions of sample collection and delivery timepoints for women who delivered full-term or preterm in the two cohorts are shown in Fig. 1. The SU cohort used for model development comprised of 19 full-term pregnancies with 222 urine samples collected in the 1st (*n* = 49) and 2nd (*n* = 173) trimesters, and 11 preterm pregnancies with 107 urine samples collected in the 1st (*n* = 24) and 2nd (*n* = 83) trimesters. The UAB cohort used for model validation had 12 full-term pregnancies with 78 urine samples collected in the 3rd trimester and 12 preterm pregnancies with 78 urine samples collected in the 1st (*n* = 5) and 2nd (*n* = 73) trimesters. Additional cohort demographics are shown in Table 1.

**Table 1:** Demographics and birth characteristics of the development and validation cohorts

	SU Cohort				UAB Cohort			
	Early PTB (*n* = 11)	Very early PTB (*n* = 2)	Full-term (*n* = 19)	P-value	Early PTB (*n* = 12)	Very early PTB (*n* = 4)	Full-term (*n* = 12)	P-value
**Maternal Age, mean ± SD**	29.6 ± 7.3	38.5 ± 9.2	32.2 ± 4.7	0.259	27.9 ± 4.4	27.5 ± 5.1	25.4 ± 4.9	0.415
**GA weeks at del, mean ± SD**	30.4 ± 4.4	22.0 ± 1.4	39.5 ± 1.2	< 0.001	28.5 ± 3.4	24.8 ± 1.3	38.0 ± 1.2	< 0.001
**Race, N (%)**				< 0.001				0.01
American-Indian	1 (9.1)	0 (0.0)	0 (0.0)		0 (0.0)	0 (0.0)	0 (0.0)	
Asian	1 (9.1)	1 (50.0)	0 (0.0)		0 (0.0)	0 (0.0)	0 (0.0)	
African-American	1 (9.1)	0 (0.0)	0 (0.0)		9 (75.0)	1 (25.0)	12 (100.0)	
Indian	1 (9.1)	0 (0.0)	0 (0.0)		0 (0.0)	0 (0.0)	0 (0.0)	
Pacific Islander	1 (9.1)	1 (50.0)	0 (0.0)		0 (0.0)	0 (0.0)	0 (0.0)	
White	4 (36.4)	0 (0.0)	19 (100.0)		2 (16.7)	2 (50.0)	0 (0.0)	
Other	2 (18.2)	0 (0.0)	0 (0.0)		1 (8.3)	1 (25.0)	0 (0.0)	
**Body mass index**,** mean ± SD**	27.7 ± 5.4	30.9 ± 1.9	22.4 ± 3.1	0.003	30.9 ± 10.3	28.9 ± 9.9	28.3 ± 7.3	0.778
**Delivery mode**,** n (%)**				< 0.001				0.006
C-Section	8 (72.7)	0 (0.0)	6 (31.6)		5 (41.7)	3 (75.0)	0 (0.0)	
NSVD	3 (27.3)	2 (100.0)	12 (63.2)		7 (58.3)	1 (25.0)	12 (100.0)	
OVD	0 (0.0)	0 (0.0)	1 (5.3)		0 (0.0)	0 (0.0)	0 (0.0)	
**History of preterm del**,** n (%)**				0.042				0.619
No	5 (45.5)	0 (0.0)	16 (84.2)		0 (0.0)	0 (0.0)	2 (16.7)	
Yes	6 (54.5)	2 (100.0)	3 (15.8)		12 (100.0)	4 (100.0)	10 (83.3)	

Values are mean ± SD or numbers (percentages). SD: Standard deviation; IQR: Interquartile range; NSVD: Normal spontaneous vaginal delivery; OVD: Operative vaginal delivery. Del: deliveries

### Ethics approval and consent to participate

The study was approved by ethics committees at Stanford University and the University of Alabama, Birmingham, and written informed consents were obtained from all participants. All methods were carried out in accordance with relevant guidelines and regulations.

### Urinary metabolite extraction and global liquid chromatography mass spectrometry (LC-MS) analysis

As previously described [22], urinary metabolites were extracted using a protein precipitation-based approach and then subjected to LC-MS/MS global metabolomic profiling analyses. 10 µL of urine was extracted with 100 µL of methanol containing 5 µg/mL of ^13^C_5_, ^15^N-l-proline, ^13^C_6_-l-arginine, and D_5_-l-glutamine. These externally spiked exogenous isotope-labelled metabolites were used as references for sample preparation and extraction efficiency. The extract was vortexed for 1 min and centrifuged at 12,000×g for 5 min. 90 µL of supernatant was then collected for the global metabolomics analyses.

The QC sample was generated by pooling a mixture of 10 µL of each of the full-term and preterm urine samples into a single tube [28]. Samples from full-term and preterm pregnancies as well as QC samples were injected into the MS. For batch analyses, QC urine samples were analyzed repetitively at a frequency of one QC injection per 10 testing samples to allow for systematic assessments of the data quality. Urine samples were injected using a LC metabolomics platform consisting of hydrophilic interaction chromatography (HILIC) and global MS analysis using a Vanquish UHPLC system coupled to a Q Exactive plus and Q Exactive HF hybrid Quadrupole-Orbitrap mass spectrometers (ThermoFisher, San Jose, CA).

### Data preprocessing and statistics

As previously described [22], data preprocessing was performed to convert the MS raw data into a data matrix of relative abundance of metabolites among all samples. The obtained features were normalized by a robust QC-based locally estimated scatterplot smoothing (LOESS) signal correction approach, and each feature was independently corrected by fitting a LOESS curve to the signal response measured in QC replicates. This procedure was done using an R xcms package. Metabolite values in each sample were then normalized by the median values measured with QC samples to reduce any batch effects. Further normalization was performed using a probabilistic quotient normalization method [29].

Metabolomic features with coefficient variation (CV) ≤ 20% in QC and missing values in ≤ 30% of samples were selected for downstream analyses. Differential expression (DE) was performed using DESeq2 [30]. The Gini Index (Gini Impurity) is essentially the probability of a new record being incorrectly classified at a given node in a decision tree, based on the training data. Through weighted by the proportion of samples reaching that node in each individual decision tree, the Gini Importance (the mean of a feature’s total decrease in Gini Index) could be calculated. This is effectively a measure of how important a feature is for estimating the value of the target variable across all the trees. A higher mean decrease in Gini indicates higher feature importance [31, 32]. Therefore, metabolites that were significantly associated with PTB were selected using Gini Importance based on the impurity reduction of splits. Partial least-squares discriminant analysis (PLS-DA) was performed to characterize metabolomic profiling of samples collected at the 1st and 2nd trimesters. KEGG (Kyoto Encyclopedia of Genes and Genomes) pathway database [33] was used to identify the metabolic pathways associated with PTB. The P-value of the metabolic pathway was calculated by the one-sided Fisher exact test and corrected using false discovery rate to control the false positive rate of the analysis results. And the enrichment value of each metabolic pathway was calculated as the ratio of the number of differentially expressed genes to the number of genes annotated to that pathway. The P-Value (less than 0.05) and enrichment value were combined to determine the significant enrichment pathways [34].

All statistical analyses were preformed using R packages, mainly including pROC [35], ropls [36], mlr [37], clusterProfiler [38], survival [39].

### Metabolite identification and metabolite-based modeling

Metabolite identification (ID) was performed as a tier 1 or 2 ID with chemical standards according to MSI [40]. With tandem mass spectrometry (MS/MS, Thermo Q Exactive plus) data of urine samples and manual review confirmation, the generated MS1/MS2 profiles were searched in public databases: HMDB (http://www.hmdb.ca/), MoNA (http://mona.fiehnlab.ucdavis.edu/), MassBank (http://www.massbank.jp/), METLIN (https://metlin.scripps.edu), and NIST (https://www.nist.gov/). The reference compounds of the metabolites of interest were procured and subjected to a tier one ID comparing retention times and MS1 and MS2 patterns with biomarker candidates using the same LC-MS/MS protocol.

The XGBoost models were developed to predict risk for early and very early PTB deliveries using the metabolic features, of which structure IDs were determined. All pregnancies were risk-stratified into categories of low- and high-PTB risks at the time of urine serial samplings. To evaluate model performance, receiver operating characteristic curves (ROCs) and area under the curve (AUCs) were used to evaluate prediction performance. Multivariable Cox regression was performed for subpopulation comparison. Based on the observed sample statistics after the predictive model grouping, Kaplan-Meier survival analysis was adopted to calculate the probability that the pregnant woman will be able to continue the pregnancy in each gestational week by the multiplication theorem of probabilities [41]. The continuous curves with the gestational week as the horizontal axis and the probability of continuous pregnancy as the vertical axis were drawn to illustrate the relationship between the gestational week and the ongoing pregnancy rate in different PTB risk groups. Through the survival analysis method, the probabilities of delivery in different gestational week could be compared between PTB low and high-risk categories to further validate the performance of the predictive models.

## Results

### A unique PTB-associated metabolomic pattern and metabolic pathway analyses

**Fig. 2:**
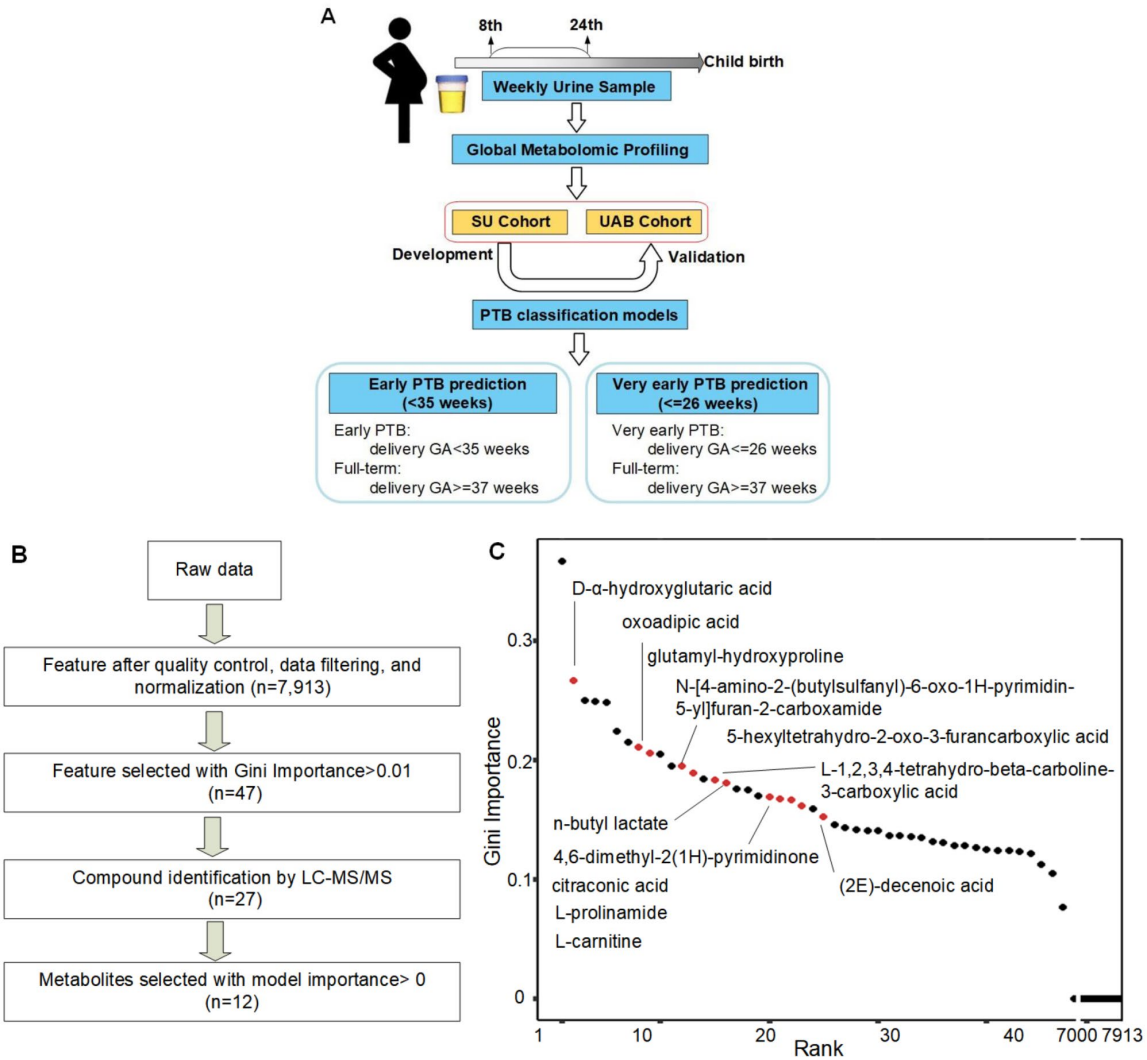
Study workflow and selection of the significant metabolic features associated with PTB outcomes. (**A**) Schematic diagram of the overall study workflow. Metabolite-based models predictive of early and very early PTB outcomes were established using urine samples from the SU and UAB cohorts. (**B**) The workflow of data preprocessing and feature selection. (**C**) LC-MS/MS-derived metabolite features ranked by the Gini Importance. Names of the 12 selected metabolites (red circles, with tier 1 or 2 structure ID) are shown

**Fig. 3:**
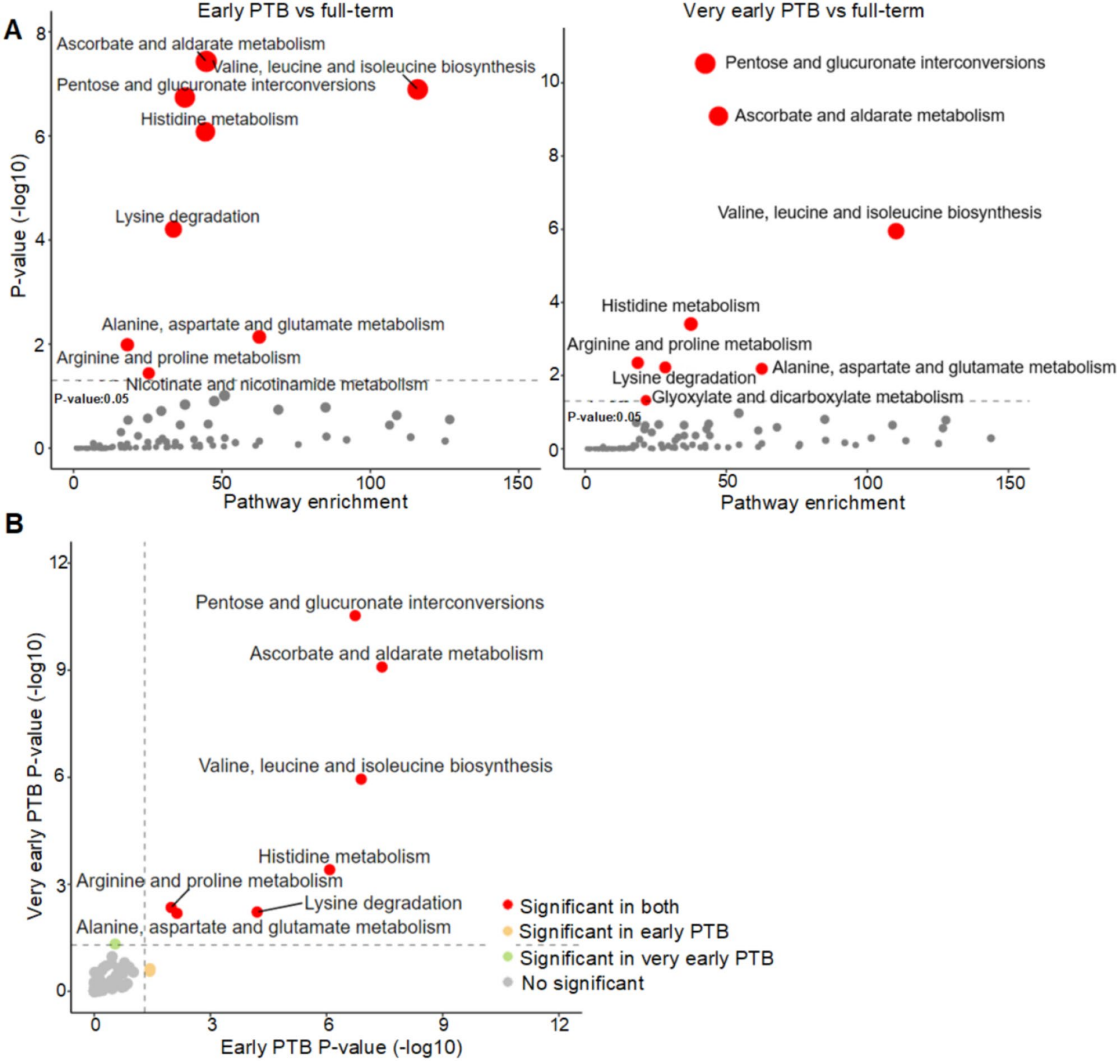
KEGG metabolic pathway enrichment analyses comparing maternal urine metabolomes of early and very early preterm with full-term pregnancies. (**A**) The vertical axis (y-axis) displays the − log10(P-value) of metabolic pathways comparing early and very early preterm delivery with full-term pregnancies, and the horizontal axis (x-axis) displays the corresponding pathway enrichment. Pathways with *P* < 0.05 are shown as red circles. (**B**) Comparison of metabolic pathway enrichment in early and very early preterm delivery analyses. Pathways that are significant in both are shown as red circles

The study workflow is as diagramed in Fig. 2A. Outlined in Fig. 2B, after QC, data filtering, and normalization, a total of 7,913 metabolic features were identified by LC-MS metabolomic profiling of the SU cohort. These features were examined globally with unsupervised clustering algorithm (PLS-DA, Supplemental Fig. 1), revealing unique metabolomic patterns in full-term or preterm pregnancies in the 1st and 2nd trimesters. Pathway enrichment analyses revealed significant pathways (*P* < 0.05) associated with early and very early preterm deliveries (Fig. 3A). Both share similar significant metabolic pathway enrichment (Fig. 3B): pentose and glucuronate interconversions; ascorbate and aldarate metabolism; valine, leucine, and isoleucine biosynthesis; histidine metabolism; arginine and proline metabolism; lysine degradation; and alanine/aspartate/glutamate metabolism.

### PTB predictive metabolite biomarkers

**Fig. 4:**
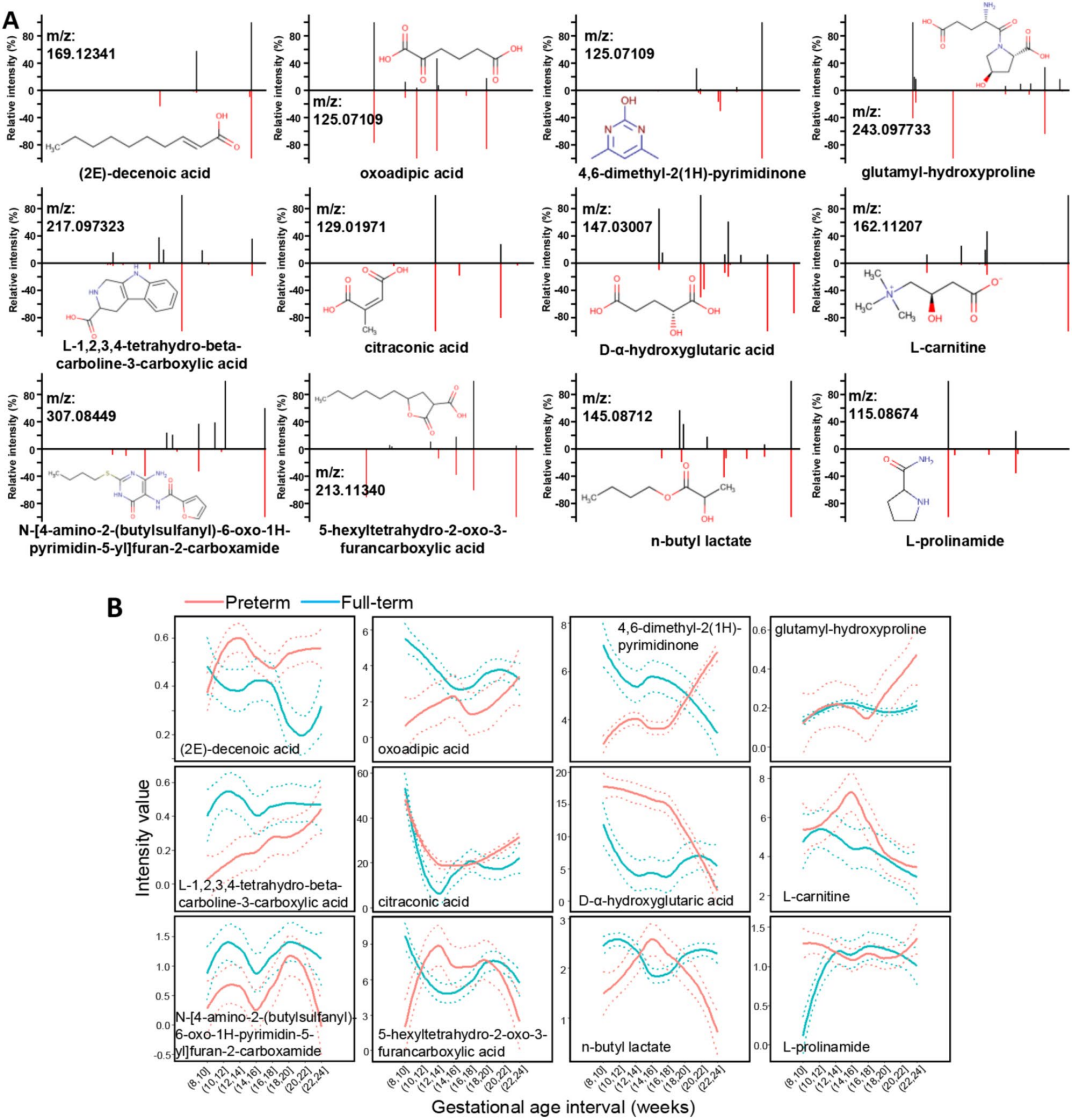
Characterization of the metabolite biomarkers. (**A**) The structural identification of the 12 metabolites by MS/MS fragmentation against reference compound standards. Measured MS/MS spectral fragmentation profiles (top, black lines) matching chemical standards (bottom, red lines). (**B**) PTB metabolite biomarkers with unique urinary abundance patterns along gestation. The mean levels (solid lines) with the 95% CIs (dotted lines) of the metabolite changes against the gestational progression of the 8th and 24th weeks are shown for preterm (red) and full-term (blue) births

Analyses of the Gini Importance of metabolic features (Fig. 2C) comparing early PTB with full-term urinary metabolomes revealed 47 maternal metabolite features (Gini Importance > 0.01) significantly associated with early preterm deliveries. 27/47 compounds were structurally identified by LC-MS/MS profiling and reference compound matching analyses. To develop a metabolic panel predictive of early outcomes, XGBoost multivariate modeling analysis was performed. 12/27 compounds were found to have a model importance > 0 (Supplemental Fig. 2): L-prolinamide, 4,6-dimethyl-2(1 H)-pyrimidinone, citraconic acid, n-butyl lactate, D-α-hydroxyglutaric acid, oxoadipic acid, L-carnitine, (2E)-decenoic acid, 5-hexyltetrahydro-2-oxo-3-furancarboxylic acid, L-1,2,3,4-tetrahydro-beta-carboline-3-carboxylic acid, glutamyl-hydroxyproline, and N-[4-amino-2-(butylsulfanyl)-6-oxo-1 H-pyrimidin-5-yl]furan-2-carboxamide. Figure 4A illustrates the confirmation of these 12 metabolites with reference compound standards by MS/MS analyses. When comparing full-term vs. preterm samples, unique and differentiating GA patterns of these 12 metabolites were observed (Fig. 4B).

### Performance of the early PTB prediction model

The 12-maternal-urine-metabolite-based models developed were used to predict the two subsets of PTB from those full-term pregnancies. In Supplemental Fig. 3A, the 12-compound panel can predict early PTB with an AUC of 0.995 for the SU training cohort and 0.964 for the UAB testing cohort. At a sensitivity of 100% (for SU) or 97.4% (for UAB), our early preterm delivery risk predictive model achieved a PPV of 88.4% and 88.4% for SU and UAB, respectively (Supplemental Table 1). Supplemental Fig. 3B shows that the 12-compound panel can predict very early preterm delivery with AUC of 0.950 and 0.830 for SU and UAB cohorts, respectively. At a sensitivity of 95% (for SU) and 60% (for UAB), our very early preterm delivery predictive model achieved a PPV of 48.7% and 56.2% for SU and UAB, respectively.

We performed a Kaplan-Meier analysis to identify the delivery dates for pregnancies predicted to be at risk of early or very early preterm deliveries (Supplemental Fig. 4). When comparing GA at delivery between low and high-risk pregnancies, a significant shift was observed with high-risk pregnancies delivering significantly earlier (*P* < 0.001 for early preterm delivery prediction in either SU or UAB cohorts) during gestation. A similar trend was observed for the very early preterm delivery predictors when compared with deliveries at term.

## Discussion

### Summary of main findings

We recruited pregnant women from two centers in the US to explore the ability of the women’s pregnancy urinary metabolome to predict early premature birth (< 35 weeks’ GA) or very early premature birth (≤ 26 weeks’ GA) from full-term pregnancies. Among the global metabolomic features obtained from the original serially collected urine metabolomics profiling, 12 urinary metabolites were identified predictive of early preterm delivery outcomes. Based on these metabolites, we established early as well as very early preterm delivery risk prediction models using the urine samples collected in the 8th to 24th weeks of pregnancy. Our two models were validated to effectively predict PTB risks in racially diverse pregnancies (primary group: SU, White; UAB, African-American). These results support the hypothesis that longitudinal analysis of urine metabolites of a women during pregnancy can predict the occurrence of an early or a very early preterm delivery and provide an effective no-invasive and low-cost PTB detection method.

### Comparison with prior work

This current work is an extension of our previous findings [22] that the weekly baseline profile of the human pregnancy metabolome provides a high-resolution molecular reference for future studies of adverse pregnancy outcomes. In terms of predictors, sampling matrices, and test methods, our PTB risk prediction models were different from previous studies [9, 10, 19, 31, 42–44]. With such differences and with so few studies on pregnant women using urine samples collected weekly beginning from the 1st trimester to delivery, direct comparisons to the extant literature are not informative.

### PTB biomarker biological implications

Women’s urine metabolite biomarkers significantly associated with the early or very early preterm delivery outcomes were identified. Comparing our urinary biomarker findings in normal gestational dating [22] and PTB outcomes, oxoadipic acid, L-carnitine, and glutamyl-hydroxyproline were revealed to be overlapping biomarkers. D-α-hydroxyglutaric acid (DGA) is the biochemical hallmark of patients affected by neurometabolic disorders [45]. Ascorbate and aldarate metabolism was identified as significantly enriched in both the early and very early preterm birth metabolomics analyses. This is in line with previous findings that ascorbic acid deficiency, as a result of the low intake of vitamin C, may lead to premature rupture of the membranes [46]. Arginine and proline metabolism is a common pathway significantly enriched with PTB outcomes in both our previous blood [21] and current urinary metabolomic analyses. L-1,2,3,4-tetrahydro-beta-carboline-3-carboxylic acid is the most likely to be related to smoking metabolites, particularly those associated with nicotine metabolism. Beta-carbolines are compounds that can be formed during the combustion of tobacco and are also endogenous metabolites. They have structural similarities to nicotine metabolites and can interact with nicotinic acetylcholine receptors [47, 48]. This metabolite, derived from tryptophan metabolism, shows notably higher predictive importance for very early PTB compared to early PTB (Supplemental Fig. 2). The enhanced predictive power for very early PTB may reflect underlying biological differences in these distinct phenotypes. L-1,2,3,4-tetrahydro-beta-carboline-3-carboxylic acid, being involved in tryptophan and neurotransmitter metabolism, might be particularly sensitive to the more pronounced metabolic perturbations characteristic of very early PTB. These observations provide evidence to support that the underlying pathophysiology of impaired metabolic pathways, including arginine, can compromise a pregnancy and affect fetal programming.

### Advantage of urine testing of PTB risks

This study highlights the clinical utility of urinary metabolomic profiling in pregnant women, particularly for predicting early or very early preterm birth (PTB). Through the identification of a urine metabolite panel, it becomes possible to develop a noninvasive test that may serve as an alternative to ultrasound for assessing fetal growth. This approach could benefit women with limited ultrasound access by offering a simple, high-resolution method to monitor and manage high-risk pregnancies. While PTB may not always be preventable, early identification allows for timely interventions, including potential moderate delays in delivery (1-2 weeks) or facilitating hospital admissions to ensure safe delivery.

Our longitudinal findings demonstrate the promise of urinary metabolomic profiling as a tool for identifying biomarkers associated with pregnancy complications, particularly PTB. Translating these discovery-phase biomarkers into clinical practice, however, presents unique challenges. For instance, traditional antibody-based assays may not be practical for a panel of small-molecule metabolites, necessitating the use of quantitative mass spectrometry, which, despite its advantages, remains limited in clinical settings.

The next steps involve carefully designed prospective validation studies that evaluate these biomarkers using both mass spectrometry and antibody-based methods, where feasible, to ensure clinical applicability. With its noninvasive sampling and molecular precision, urinary metabolomics has the potential to significantly improve diagnostic and therapeutic strategies in pregnancy care, providing a practical and innovative approach to managing complications in pregnancy.

### Limitations

This study has several limitations. First, as a retrospective analysis, it includes participants from two distinct geographic regions (California and Alabama), resulting in disparities in race and age distributions between cohorts. The uneven representation of racial groups across our discovery and validation cohorts restricts our ability to draw robust, generalizable conclusions about PTB risk across racial groups. Underrepresentation of some racial groups limits our capacity to detect race-specific metabolic signatures or to confirm marker consistency across populations. Future studies should prioritize balanced racial representation through multi-center recruitment and targeted enrollment, allowing a more comprehensive evaluation of how racial background influences metabolic profiles and PTB risk, ultimately supporting the development of equitable diagnostic approaches. Second, while our model can classify early or very early preterm delivery (high and low risk) based on a single urine test during pregnancy, we have not investigated time to delivery, which may affect the model’s clinical utility. Third, the absence of detailed phenotype classifications for PTB limits our analysis of specific pathological characteristics associated with the identified biomarkers. Although this study does not directly address PTB etiology, future research will investigate the pathophysiology linked to these biomarkers, their associated metabolic pathways, and potential correlations with specific PTB phenotypes. Fourth, we did not collect maternal smoking status, despite its known associations with adverse pregnancy outcomes. This omission is a limitation in our study design, and future research should include smoking status data to strengthen our analysis. Fifth, the lack of socioeconomic status (SES) data represents another limitation. SES may act as a significant confounder, affecting pregnancy outcomes through lifestyle factors such as diet, environmental exposures, and access to prenatal care. Including SES data in future studies will help determine whether the identified metabolic signatures are robust across socioeconomic backgrounds and ensure that any developed screening tools are effective for diverse populations. Sixth, fetal sex was not included in the current model, which is another potential limitation. Emerging evidence suggests fetal sex may influence maternal metabolism and pregnancy complications, including PTB risk. Future research will conduct a stratified analysis by fetal sex to explore any sex-specific patterns in the predictive signatures and assess if model performance varies based on fetal sex. Finally, a larger prospective cohort study across diverse racial, ethnic, and socioeconomic backgrounds is necessary to further validate the clinical utility of our maternal urinary metabolite panel for PTB prediction.

## Conclusions

Our study demonstrates that maternal urinary metabolomic profiling is a noninvasive, highly sensitive, and accurate method for predicting risk of early preterm delivery (< 35 weeks’ GA) or very early preterm delivery (≤ 26 weeks’ GA). in the 1st or 2nd trimester. The robustness of modeling with diverse racial groups and maternal ages, and the simplicity of sample acquisition add to its potential for clinical development and use. This prediction tool provides an effective reference for the diagnosis of PTB and enables targeted early intervention to ensure the safety of pregnant women and fetus. By following with the metabolite biomarkers and underlying enriched pathways, we may shed new light on the mechanism of action underlying the pathophysiology related to abnormal fetal development and pregnancy disorders including spontaneous PTB.

## Electronic supplementary material

Below is the link to the electronic supplementary material.

Supplementary Material 1: Supplemental Figure 1: Distribution of individual samples in partial least-squares discriminant analysis based on 7,913 features as a function of the PTB outcomes in either 1st or 2nd trimester, re-spectively. The two orthogonal components with most of the inertia are shown.

Supplementary Material 2: Supplemental Figure 2: The importance of the 12 metabolites in early and very early PTB risk models.

Supplementary Material 3: Supplemental Figure 3: Evaluation of early and very early PTB prediction with SU and UAB co-horts. (A) Area under the curves (AUCs) and confusion matrix performance of early PTB prediction model. (B) AUC and confusion matrix performance of very early PTB prediction model.

Supplementary Material 4: Supplemental Figure 4: Kaplan-Meier analyses of deliveries contrasting the low- and high-risk PTB pregnancies. (A) Early preterm delivery prediction model. (B) Very early preterm delivery prediction model. The vertical axis (y-axis) displays the probability of ongoing pregnancy, and the horizontal axis (x-axis) displays the gestational age in different PTB risk groups. The corresponding table provides the specific individual number who can still be in an ongoing pregnant state at different gestational age weeks.

Supplementary Material 5: Supplemental Table 1: Sensitivity, specificity, positive predictive value (PPV) and negative pre-dictive value (NPV) together with the 95% CIs in different models. SU, Stanford Hospital and Clinics; UAB, University of Alabama.

## Data Availability

The datasets used or analyzed during the current study are available from the corresponding author on reasonable request.
