# Supplementary figures and images for: Prediction of risk for early or very early preterm births using high-resolution urinary metabolomic profiling
Source: BMC Pregnancy Childbirth. 2024 Nov 25;24:783. doi: 10.1186/s12884-024-06974-2 (PMC11587579; doi:10.1186/s12884-024-06974-2)

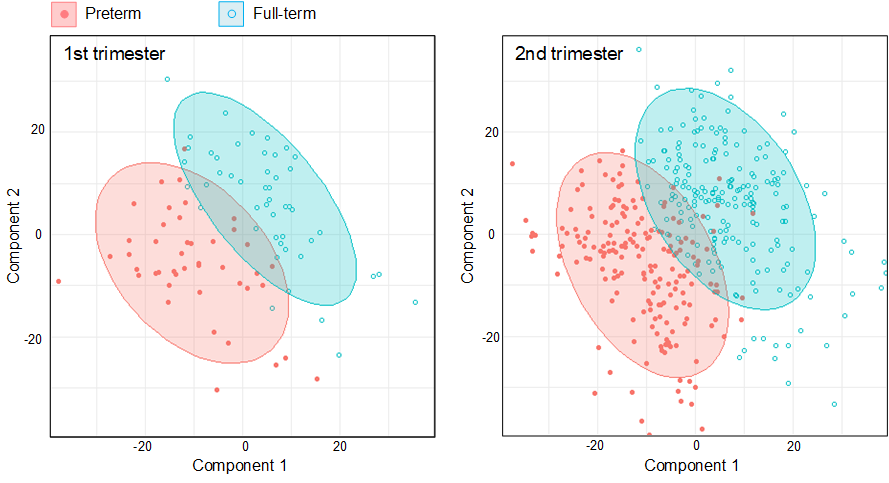

Supplement: Supplementary file 1 — Supplementary Material 1: Supplemental Figure 1: Distribution of individual samples in partial least-squares discriminant analysis based on 7,913 features as a function of the PTB outcomes in either 1st or 2nd trimester, re-spectively. The two orthogonal components with most of the inertia are shown. [file 12884_2024_6974_MOESM1_ESM.png]

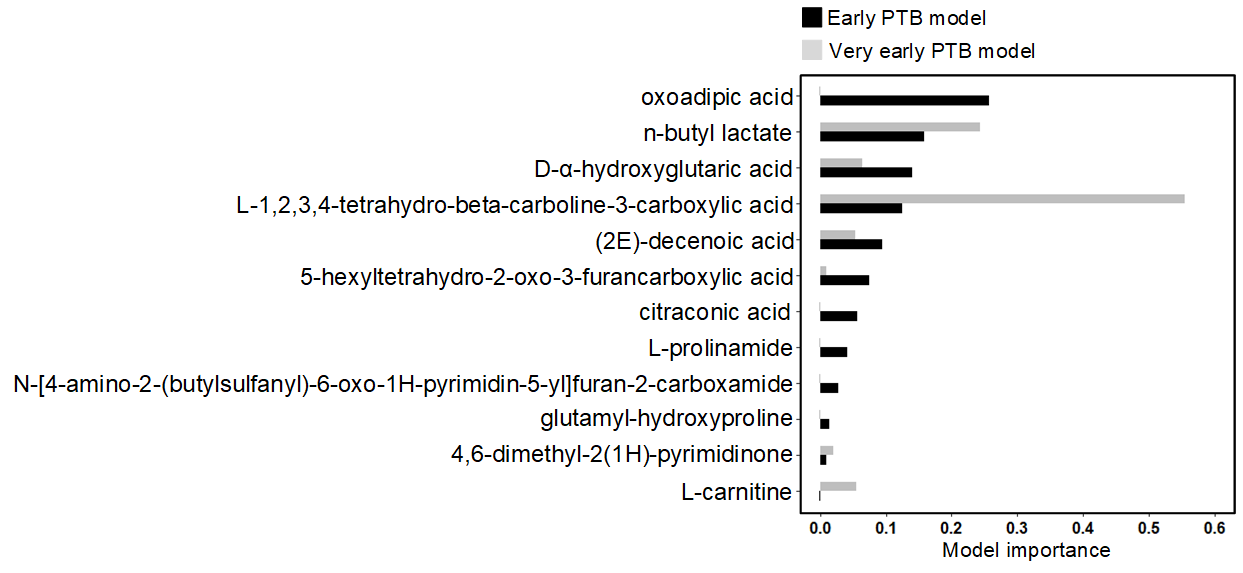

Supplement: Supplementary file 2 — Supplementary Material 2: Supplemental Figure 2: The importance of the 12 metabolites in early and very early PTB risk models. [file 12884_2024_6974_MOESM2_ESM.png]

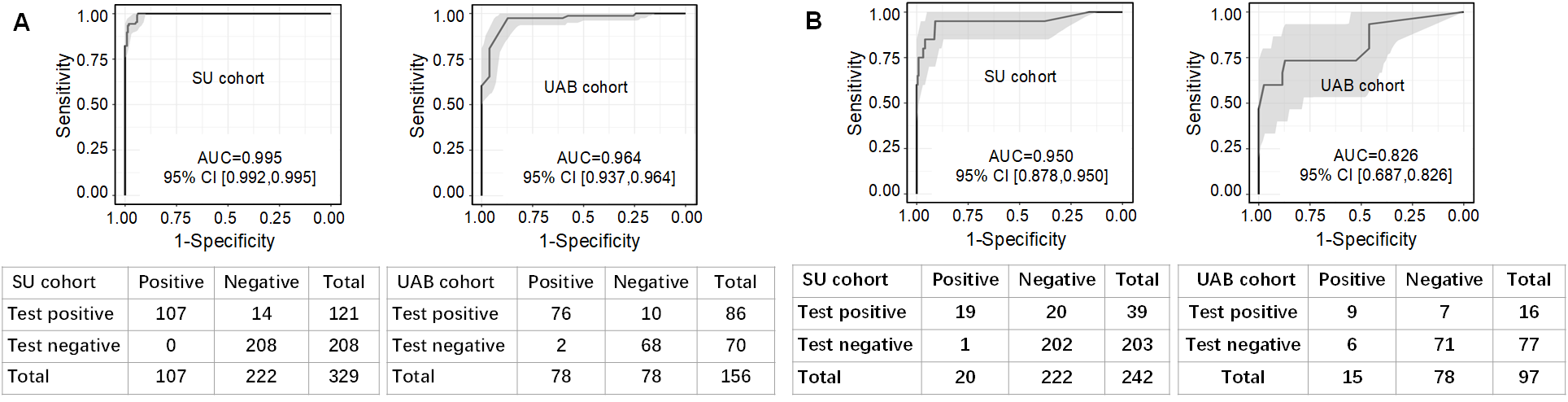

Supplement: Supplementary file 3 — Supplementary Material 3: Supplemental Figure 3: Evaluation of early and very early PTB prediction with SU and UAB co-horts. (A) Area under the curves (AUCs) and confusion matrix performance of early PTB prediction model. (B) AUC and confusion matrix performance of very early PTB prediction model. [file 12884_2024_6974_MOESM3_ESM.png]

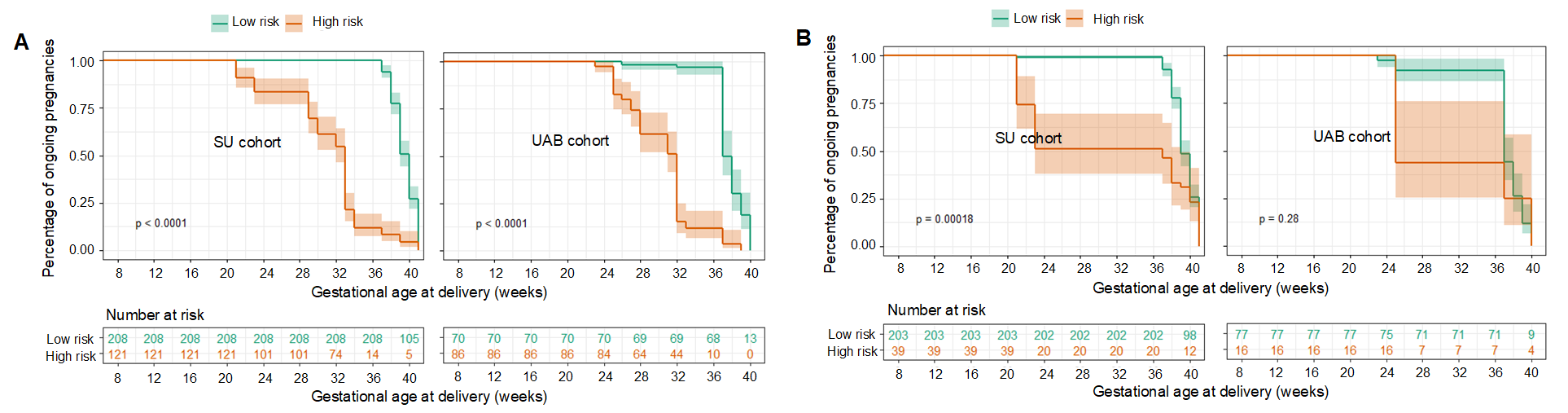

Supplement: Supplementary file 4 — Supplementary Material 4: Supplemental Figure 4: Kaplan-Meier analyses of deliveries contrasting the low- and high-risk PTB pregnancies. (A) Early preterm delivery prediction model. (B) Very early preterm delivery prediction model. The vertical axis (y-axis) displays the probability of ongoing pregnancy, and the horizontal axis (x-axis) displays the gestational age in different PTB risk groups. The corresponding table provides the specific individual number who can still be in an ongoing pregnant state at different gestational age weeks. [file 12884_2024_6974_MOESM4_ESM.png]
