# Supplementary material for: Prediction of risk for early or very early preterm births using high-resolution urinary metabolomic profiling
Source: BMC Pregnancy Childbirth. 2024 Nov 25;24:783. doi: 10.1186/s12884-024-06974-2 (PMC11587579; doi:10.1186/s12884-024-06974-2)
Supplement: Supplementary file 5 — Supplementary Material 5: Supplemental Table 1: Sensitivity, specificity, positive predictive value (PPV) and negative pre-dictive value (NPV) together with the 95% CIs in different models. SU, Stanford Hospital and Clinics; UAB, University of Alabama. [file 12884_2024_6974_MOESM5_ESM.docx]

**Supplemental Table 1.** Sensitivity, specificity, positive predictive value (PPV) and negative predictive value (NPV) together with the 95% CIs in different models. SU, Stanford Hospital and Clinics; UAB, University of Alabama.

| Model | Cohorts | Number | Sensitivity 95% CI | Specificity 95%  CI | NPV | PPV |
| --- | --- | --- | --- | --- | --- | --- |
| Early PTB prediction | SU | 329 | 100% (107/107) 100% - 100% | 93.7% (208/222) 90.5% - 96.8% | 100% (208/208) | 88.4% (107/121) |
|  | UAB | 156 | 97.4% (76/78) 93.6% - 100% | 87.2% (68/78) 79.5% - 93.6% | 97.1% (68/70) | 88.4% (76/86) |
| Very early PTB prediction | SU | 242 | 95% (19/20)  85% - 100% | 91% (202/222) 87.4% - 94.6% | 99.5% (202/203) | 48.7% (19/39) |
|  | UAB | 80 | 60% (9/15)  33.3% - 86.7% | 91% (71/78) 84.6% - 97.4% | 92.2% (71/77) | 56.2% (9/16) |
